# Antibiotics-Related Adverse Drug Reaction in a Tertiary Hospital in Saudi Arabia: A Cohort, Retrospective Study

**DOI:** 10.7759/cureus.38431

**Published:** 2023-05-02

**Authors:** Hanan Ahmed Bakri, Abdulaziz A Jaly

**Affiliations:** 1 Clinical Pharmacy, King Fahd Central Hospital, Jazan, SAU; 2 Pharmacy, Jazan University Hospital, Jazan, SAU

**Keywords:** rashes., naranjo scale, cephalosporins, antibiotics, adverse drug reactions

## Abstract

Introduction: Adverse drug reactions (ADR) are caused by a wide range of drugs including antibiotics. Currently, the prevalence and pattern of antibiotic-related ADR in Saudi Arabia are not well reported. The present study aimed to evaluate the ADR pattern caused by antibiotics in a tertiary healthcare center.

Methods: This was a retrospective study conducted on 85 patients admitted to tertiary care hospital medical wards during the period from 2015 to 2019. The following data such as patient demographics (age, gender, weight, height), reason for admission, number of antibiotics use, comorbid condition, antibiotic(s) involved in ADR, classification of ADR, and type of ADR were recorded. Naranjo’s scale was used to measure the probability of ADR.

Results: Among the 85 patients, the most frequent type of antibiotic was cephalosporins in 36.47%, followed by penicillins in 31.76% of the patients. The major type of ADR was rash (52.95%), followed by anaphylaxis reactions (10.59%) of the patients. Based on the Naranjo scale, the ADR was possible in 80% and probable in 18.82% of the cases. The presence of medical conditions displayed a significant association with the development of rashes (p=0.03). In addition, the female gender (p=0.009) and the presence of medical conditions (p=0.03) showed significant association with the development of anaphylaxis.

Conclusion: Cephalosporin and penicillins were the most common antibiotics responsible for ADR, and the rash was the most common ADR.

## Introduction

Globally, adverse drug reaction (ADR) is a frequently encountered treatment-related complication by individuals, physicians as well as pharmacists and it causes significant morbidity and mortality among patients [1]. As per the World Health Organization (WHO), ADR is defined as a harmful, unintended reaction to therapeutic agents that occurs at normal doses during the treatment for any medical conditions or diseases, diagnosis, and modifications of any physiological functions [2]. Prevention of ADR is possible after analyzing the patient's medication history in detail and with proper follow-up so that the physician can understand the patient's prior and current experience during the drug treatment [3]. The occurrence of ADR depends on many factors such as drugs, infections, social behavior, and individuals in low-income settings. In addition, the physiological and illness condition of the patients also contributes to ADR. Further, the ADR response is more profound in children and elderly as compared to adult patients and this might be due to the variation in the metabolism and excretion functions and also reduced functional reserve during advanced age [4]. According to the Global Burden of Disease Study 2017, the worldwide incidence of ADR due to medical procedures is 438.97/100,000 population with an annual incidence of 32.9 million people affected [5]. A recent systematic review and meta-analysis study showed a prevalence of ADR at 8.32%, preventable ADR at 12.35%-37.96%, and an overall rate of 22.96% in primary healthcare settings [6]. In Saudi Arabia (KSA), data from various hospitals displayed an overall ADR incidence of 6.1/100 admissions, and for preventable ADR the rate is 16.9/100 admissions [7].

Several studies show that there has been a rampant increase in the use of antibiotics with a shift towards administering broad spectrum in hospitals [8]. In addition, misuse or self-medication of antibiotics also leads to antibiotic resistance and is associated with more ADRs such as skin allergy, organ toxicity, and additional infection due to resistant organisms. The global estimate of antibiotic-associated ADR is not clearly reported, and the incidence varies USA (20%) [9], South Korea (62.8%) [10], Uganda (19%) [11], and India (40.9%) [12]. Previous studies evaluating the antibiotic-associated ADR have certain limitations such as ADR evaluation in in-hospital settings or using hospital databases but not measuring after discharge and only considering ADR associated with a single class of antibiotics [13]. In KSA, numerous reports have been published for ADRs but only a few studies focused on antibiotics. Therefore, the present study was conducted to evaluate the incidence of antibiotic-associated ADR, frequency of antibiotics, type of antibiotics, and risk factors for ADR in patients undergoing antibiotic therapy at tertiary care health centers in KSA.

## Materials and methods

Setting and study design

This was a retrospective cohort study conducted on patients attending Security Forces Hospital, a tertiary healthcare hospital in Riyadh, Saudi Arabia, from 2015 to 2019. In this study, among the hospitalized patients the ADR related to antibiotics was recorded (through medical electronic files of patients) and included for the analysis. The analysis also included the ADRs that patients experienced after receiving antibiotics and other drugs.

This study was approved by the Institutional Review Board (IRB) of Security Forces Hospital and proper ethical approval was obtained before the initiation of the study (IRB Approval number: HP-01-R079). The data collection form includes patient demographics (age, gender, weight, height), reason for admission, number of antibiotics use, comorbid condition, antibiotics involved in ADR, classification of ADR, and type of ADR.

Inclusion and exclusion criteria

All hospitalized patients with documented antibiotics-related ADR (medical, surgical, adult, pediatrics, or neonates) were included in this study. Any drug allergy or adverse effect will give a notification on the patient's file and a report of the incident (ADR or allergies) will be documented and included. Other ADR cases caused by any medications other than antibiotics as well as patients who developed antibiotics-related ADR and were admitted to the hospital for < 24 hours were excluded.

A total of 375 patients with ADR or allergy from antibiotics were initially screened according to inclusion criteria of the study plan and in that, only 85 patients have a complete report or documentation for that adverse effect. ADR probability was reported according to the Naranjo scale [14]. Naranjo algorithm, the most frequently used scale to evaluate ADR causes. This scale encompasses 10 simple questions that are answered as yes, no, or do not know in the following areas: the temporal relationship, the pattern of reaction, dechallenge or the administration of an antagonist, rechallenge, substitute reasons, placebo reactions, the drug range in body fluids or tissue, the dose-response correlation, the patient’s prior knowledge with the drug and authorization via other objective indication. The answer to each question receives a score. The overall total score varies from 4 to 13. A score of 9 or more designates a certain ADR, a score of 5 to 8 designates a feasible ADR, a score of 1 to 4 designates a probable ADR, and an uncertain if 0 or less.

Data analysis

Descriptive analysis was done for the categorical variables and the data were represented as frequency (%). The association between ADR reactions and patient-related factors was done using Fisher’s exact test and Chi-square test. The statistical analysis was done using SPSS v 18.0 (IBM Corp., Armonk, NY).

## Results

The demographics and clinical characteristics of the study population was shown in Table 1. In total participants were selected for the study and the mean age was 29.41 ±19.44 years. Regarding age distribution, the majority of the participants were adults (19-40 years), 38.2% followed by middle age (40-65 years), 23.53% respectively. Male preponderance was observed in the present study with 58.82% being males. Chronic disease condition was present in 55.29% of the patients with diabetes, and hypertension was present in 21% of the patients. The majority of the patients were administered with single antibiotics (82.35%) followed by multiple antibiotics in 17.65% of patients.

**Table 1:** Demographics and clinical characteristics of the study population

Parameters	Frequency (%)
Age distribution	
Infants (< 1 year)	2 (2.35)
Children (> 1 – 12)	17 (20)
Teenagers (> 12 – 19)	9 (10.59)
Adults (> 19 – 40)	33 (38.82)
Middle age (40 – 65)	20 (23.53)
Elderly (> 65)	4 (4.71)
Gender	
Male	35 (58.82)
Female	50 (41.18)
Length of hospital stay (in days)	
Within a day	43 (50.59)
Within a week	22 (25.88)
More than a week	20 (23.53%)
Chronic condition	
No	38 (44.71)
Yes	47 (55.29)
Types of Chronic conditions	
Diabetes mellitus	
No	77 (90.59)
Yes	8 (9.41)
Hypertension	
No	75 (88.24)
Yes	10 (11.76)
Others	
No	41 (48.24)
Yes	44 (5.76)
Number of antibiotic used	
Single	70 (82.35)
Multiple	15 (17.65)

The distribution and type of antibiotic administration among the study participants were shown in Table 2. In this study the most frequently prescribed antibiotics was cephalosporins in 36.4% of the patients followed by penicillins in 31.76% and Quinolones in 15.29% of the patients.

**Table 2:** Distribution and type of antibiotics among the study participants

Types of antibiotics	Frequency (%)
Cephalosporins	31 (36.47)
Penicillins	27 (31.76)
Quinolones	13 (15.29)
Glycopeptides	11 (12.94)
Macrolides	4 (4.71)
Clindamycin	3 (3.53)
Carbapenems	2 (2.35)
metronidazole	2(2.35)
Sulfonamides	1 (1.18)
Tetracycline	1(1.18)
Linezolid	1(1.18)

Regarding the type of ADRs, majority of the patients had rash (52.95%), followed by anaphylaxis reactions (10.59%). The other types of ADR include ulcers of hand, foot, palate, lower lip, shortness of breathing and include 34.12%. The results were shown in Table 3.

**Table 3:** Adverse drug reaction encountered by the study participants

Adverse drug reactions	Frequency (%)
Rash	45 (52.94)
Anaphylaxis	9 (10.59)
Stevens Johnson Syndrome	2 (2.35)
Others	29 (34.12)

In this study, the present or absent of medical conditions showed significant association with the development of rashes (p=0.03). Meanwhile gender (p=0.5) and age (p=0.13) displayed non-significant association with the development of rashes during antibiotic treatment. The results were shown in Table 4.

**Table 4:** Association between rash development and demographics, clinical characteristics among the study participants # denotes Fisher’s exact test; @ denotes Chi-square test; NS - Non-significant; * significant p<0.05.

	Rash		P-value
	Present (n = 45)	Absent (n = 40)	
Age group			
Infants	2 (2.35%)	0	0.13^ # NS^
Children	13 (15.29%)	4 (4.71%)	
Teenagers	5 (5.88%)	4 (4.71%)	
Adults	13 (15.29%)	20 (23.53(	
Middle age	10 (11.76%)	10 (11.76%)	
Elderly	2 (2.35%)	2 (2.35)	
Gender			
Male	20 (23.53%)	15 (17.65%)	0.5^@ NS^
Female	25 (29.41%)	25 (29.41%)	
Medical conditions			
Present	25 (29.41%)	27 (31.76%)	0.03^@*^
Absent	20 (23.53%)	13 (15.29%)	

In addition, there was significant association between gender and anaphylaxis reaction and the incidence was higher in females and no males were affected (p=0.009). Meanwhile, during the presence of medical conditions out of nine patients, eight had anaphylaxis reactions and found to be significant (p=0.03). The results were shown in Table 5.

**Table 5:** Association between rash development and demographics, clinical characteristic among the study participants Fisher’s exact test; NS-Non significant; * significant p<0.05.

	Anaphylaxis		P-value
	Present (n = 9)	Absent (n = 76)	
Age group			
Infants	0	2 (2.35%)	0.32^NS^
Children	0	17 (20%)	
Teenagers	0	9 (10.59%)	
Adults	5 (5.88%)	28 (32.94%)	
Middle age	3 (3.53%)	17 (20%)	
Elderly	1 (1.18%)	3 (3.53%)	
Gender			
Male	0	35 (41.18%)	0.009^*^
Female	9	41 (48.24%)	
Medical conditions			
Present	8 (9.41%)	39 (45.88%)	0.03^*^
Absent	1 (1.18%)	37 (43.53%)	

The probability of ADR according to Naranjo scale was shown in Table 6. In this study, majority of the ADR was predicted to be possible in 80% and probable in 18.82% of the patients respectively. No mortality was observed in the present study.

**Table 6:** ADR probability according to Naranjo scale ADR - Adverse drug reaction

Naranjo scale	Frequency (%)
Doubtful	1 (1.18)
Possible	68 (80)
Probable	16 (18.82)

## Discussion

Mounting studies published in KSA showed that antibiotics are the most frequent cause of ADRs [15,16]. However, the majority of the data are analyzed based on the hospital medical records and from ADR reports submitted to the Saudi Food and drug authority (SFDA), but reports from tertiary healthcare centers with respect to antibiotic-associated ADR are very scarce. The present study finding underscores the antibiotic-related ADR experienced in a KSA tertiary care hospital.

In our study majority of the patients are in the age group between 19 and 40 years with 38.82% and male preponderance is observed among the study participants. Similarly, in a study conducted in KSA, more than 30% of the patients are in the age group 19-40 years, and 56.1% were reported to be males [16]. In the present study, the most common comorbid condition observed is hypertension and diabetes in 21% of the patients. Likewise, in a study conducted by Tamma et al. in the USA, the most common underlying disease condition is diabetes in 33% of the patients [9].

In this study, the most frequent type of antibiotic prescribed is cephalosporins (36.74%) followed by penicillins (31.76%). Likewise, in a study done by Junk et al. in South Korea, the most common antibiotic-associated ADR is cephalosporins (both first and second generation) in 26.1% followed by Penicillin and Quinolone in 16% of the patients [10]. In a study conducted in Jordan based on the data extracted from WHO global database (VigiBase), 48 patients are recorded for third-generation cephalosporins-mediated ADR, and the commonest antibiotic for the cause of ADR is tetracyclines (n=101), followed by fluoroquinolones (n=54) [17]. Meanwhile, in a Malaysia study, the second most common cause of ADR is a cephalosporin (20.6%) and the primary cause of ADR is mainly due to penicillins (32%) [18]. In contrast to the present study, most of the published data highlighted penicillin as a major culprit for antibiotic-associated ADR. This might be due to that penicillins are a cheap, effective, and easily available class of antibiotics prescribed for the treatment of a wide range of infections starting from respiratory infections to severe infections such as cellulitis [19]. Cephalosporins, like penicillins, are majorly responsible for immediate and delayed hypersensitivity reactions. The main difference between cephalosporins and penicillins is the six-membered dihydrothiazine ring and the presence of an R2 group [20]. Studies show that beta-lactam ring opening can lead to R2 side chain destruction and thus forms unstable conjugates and poorly fragmented identified determinants [21]. Theoretically, IgE antibodies can beta-lactam ring, the protein carrier molecule, and side chains; however, the R1 side chain and the remaining beta-lactam structure which elicit covalent binding to host proteins is the main cause for immunogenicity [22].

In this study, the major type of ADR present in the patients is rashes (52.95%) and anaphylaxis reactions (10.59%) which shows that the skin is the major organ affected. Similarly, in a study done by Arulappen et al., the skin is the most affected organ as a result of antibiotic-associated ADR in 48% of the patients [18]. In Mhaidat et al.'s study, the most common organ affected as a result of ADR is skin and subcutaneous tissue disorders in 19.48% of patients. Further, they also reported that in ADR related to skin, rashes are the most commonly observed reaction in 58 cases (10.76%) and the incidence of rashes is higher in patients consuming third-generation cephalosporins (n=11, 18.97% out of 58) [17]. In Jung et al.'s study, cutaneous and skin-related toxicities are the most common ADR event in 45.1% of cases and cephalosporin accounts for 46.25% of skin-related manifestations [10].

Moreover, the causality assessment of reported ADRs as per Naranjo’s scale revealed that most were possible (80%), then probable and limited reactions were doubtful. In Arulappen et al.'s study, 48.6% are probable and 42.3% are possible based on Naranjo’s scale ADR assessment [18]. In KSA studies based on the overall ADR, 10% are definite and the remaining ADRs are reported to be possible or probable [23].

In this study, the important factor associated with the development of rashes as a result of antibiotic-associated ADR is the presence of chronic medical conditions such as diabetes and hypertension (p=0.03). Likewise, in a prospective cohort study conducted involving four hospitals in KSA, Charlson’s comorbidity index weight showed a significant association with overall ADR (unadjusted OR; 1.25; p<0.001) [7]. Further in our study, we have observed that the female gender is associated with an increased risk of anaphylaxis reactions. Rademaker et al. showed that females had a 1.5-1.7 times greater risk of experiencing ADR as compared to males [24]. Aljadhey et al. from KSA reported that there is no significant difference in gender-based ADR incidence [7]. But in a study done by Holm et al. from Sweden, males experienced a higher rate of ADR as compared to females [25].

Strength and limitations

The major strength of the study was that it was the first to report antibiotic-associated ADRs in KSA using data from tertiary healthcare centers. The first and important limitation was it was a single-center study and the reports from various private clinics in KSA were not included. The second limitation was less sample size and there may be underreporting from physicians and registered pharmacists.

## Conclusions

In conclusion, cephalosporins and penicillins were the predominant antibiotics for the development of ADR. The most common ADR-related toxicity is skin manifestation characterized by rashes and anaphylaxis reactions. Chronic disease conditions and female gender were the significant risk factors for ADR occurrence. So, the present finding might be helpful for the SFDA and Saudi Medication Safety Center for the prevention of ADR.
